# Assessment of Cellular Responses after Short- and Long-Term Exposure to Silver Nanoparticles in Human Neuroblastoma (SH-SY5Y) and Astrocytoma (D384) Cells

**DOI:** 10.1155/2014/259765

**Published:** 2014-02-13

**Authors:** Teresa Coccini, Luigi Manzo, Vittorio Bellotti, Uliana De Simone

**Affiliations:** ^1^Laboratory of Clinical Toxicology, IRCCS Salvatore Maugeri Foundation, Scientific Institute of Pavia Medical Centre, Via Maugeri 10, 27100 Pavia, Italy; ^2^Toxicology Division, Department of Environmental Health, IRCCS Salvatore Maugeri Foundation, Scientific Institute of Pavia Medical Centre, Via Maugeri 10, 27100 Pavia, Italy; ^3^Toxicology Unit, Department of Clinical Surgical, Diagnostic and Pediatric Sciences, Faculty of Medicine and Surgery, University of Pavia, Via Maugeri 10, 27100 Pavia, Italy; ^4^Department of Molecular Medicine, Institute of Biochemistry, University of Pavia, Via Forlanini 6, 27100 Pavia, Italy

## Abstract

Silver nanoparticle (AgNP, 20 nm) neurotoxicity was evaluated by an integrated *in vitro* testing protocol employing human cerebral (SH-SY5Y and D384) cell lines. Cellular response after short-term (4–48 h, 1–100 **μ**g/ml) and prolonged exposure (up to 10 days, 0.5–50 **μ**g/ml) to AgNP was assessed by MTT, calcein-AM/PI, clonogenic tests. Pulmonary A549 cells were employed for data comparison along with silver nitrate as metal ionic form. *Short-term data*: (i) AgNP produced dose- and time-dependent mitochondrial metabolism changes and cell membrane damage (effects starting at 25 **μ**g/ml after 4 h: EC_50s_ were 40.7 ± 2.0 and 49.5 ± 2.1 **μ**g/ml for SH-SY5Y and D384, respectively). A549 were less vulnerable; (ii) AgNP doses of ≤ 18 **μ**g/ml were noncytotoxic; (iii) AgNO_3_ induced more pronounced effects compared to AgNP on cerebral cells. *Long-term data*: (i) low AgNP doses (≤1 **μ**g/ml) compromised proliferative capacity of all cell types (cell sensibility: SHSY5Y > A549 > D384). Colony number decrease in SH-SY5Y and D384 was 50% and 25%, respectively, at 1 **μ**g/ml, and lower dose (0.5 **μ**g/ml) was significantly effective towards SH-SY5Y and pulmonary cells; (ii) cell proliferation activity was more affected by AgNO_3_ than AgNPs. In summary, AgNP-induced cytotoxic effects after short-term and prolonged exposure (even at low doses) were evidenced regardless of cell model types.

## 1. Introduction

Although there is great uncertainty over the potential risks of nanotechnology products, their commercialization continues to grow albeit under conditions of uncertainty regarding the implications for health and the environment [1]. Currently, the majority of nanotechnology-enabled consumer products are based on nanoscale silver [2]. Nanosilver or nanoparticle sized silver (AgNP) provides a greater surface area of silver and theoretically a more efficacious product with higher activity than its bulk counterpart [3]. The many possibilities offered by AgNP in medical application (due to its antibacterial properties and as biosensors and imaging contrast agents), but also in the growth market of consumer products (e.g., for disinfection of water, as well as coating of surfaces in contact with human skin, textiles, or food) have raised much scientific interest and concern in the last decade [4]. The sophistication of this new nanotechnology and its proliferation (largely uncontrolled) raises new questions of health and environmental impact. One of the relevant questions regarding the safety of nanosilver in consumer products that still needs to be clarified is whether the toxic potential of nanosilver is identical to “classical” silver [5]. The point is that silver is an old problem and nanosilver is a new challenge. The scope of the new challenge is not yet clear because it is uncertain how much nanosilver is now used as an antimicrobial in commercial and consumer products and because new uses are likely to be discovered in the future. Actually, AgNP regulation is still undergoing major changes to encompass environmental, health, and safety issues [6]. The uncertainty of the safety may be compounded by lack of toxicological data and lifecycle studies of acceptable environmental exposure limits to nanosilver compounds.

Although a lot of work has been done as well as many reports and reviews have been published on nanosilver [7], few studies have been performed to address AgNP exposure and central nervous system (CNS) toxicity.

Since the extent of human exposure to AgNPs and their likelihood internalization has strongly increased during the last years, the internalized AgNPs may finally also reach the brain and encounter brain cells. Recent studies have drawn attention towards potential neurotoxic effects of AgNPs. It is well known that the CNS is highly sensitive to metals including silver [8, 9] which is known to avidly bind to the cells of nervous system. Moreover its biological half-life in the CNS is longer than that in other organs causing neurotoxic damage, consequences, and risks to the brain following prolonged exposure [10]. Decreased activity in mice and concomitant granular silver deposits, especially in brain areas involved in motor control, was found in mice orally exposed to AgNO_3_ for four months [11]. With respect to AgNPs, there is evidence, although little, indicating that AgNP itself can impact the CNS. AgNPs can cross and disrupt the rat blood-brain barrier [12, 13] and even aggravate heat stress-induced cognitive deficits, edema formation, and brain pathology [14]. *In vivo* effects of AgNPs include edema [13] and changes in the brain mRNA levels of genes linked to increased oxidative stress [15]. *In vivo* studies have also indicated that AgNPs accumulate in the rodent olfactory bulb and brain after inhalation; thus, by retrograde transport, AgNPs might get access to selective regions of the CNS inducing brain inflammation and neurotoxicity [16].

Currently, few are also the data regarding the *in vitro* neurotoxicity of AgNPs on human nervous system cells [17, 18]. The majority of toxicological data published on AgNPs derives from *in vitro* studies which typically have used rat neuronal-like cell lines, mostly PC-12 cells [19–23] or primary neural cells obtained from rodents [24–27]. Disruption and inflammation have also been demonstrated in *in vitro* blood-brain barrier (BBB) models (from rat) following incubation with AgNPs [28, 29].

Standard toxicological tests are still needed to be performed to assess the risk of AgNPs. For example, biosafety of ENMs (man-made particles with any external dimension between 1 and 100 nm) could be evaluated by tests examining general toxicity, target organ toxicity, and biocompatibility in line with regulatory requirements, applying alternative test methods (e.g., *in vitro* cellular assays) limiting the use of lab animals in toxicological research [30–32], to identify molecular endpoints and multiple toxicity pathways. *In vitro* studies can obtain toxicological data relevant to design appropriate exposure concentrations and define critical health endpoints to be monitored *in vivo*.

The present investigation proposes an integrated *in vitro* testing protocol for the screening of AgNP neurotoxicity using representative human cerebral cell lines and a battery of cytotoxicity tests to simulate both short- and long-term exposure. In particular, increasing concentrations of critical doses of an AgNP model (20 nm) have been evaluated:

- in brain cells, namely, human astrocytoma D384 and neuroblastoma SH-SY5Y cell lines, as well as in human lung epithelial cells (A549), for data comparison, since some cytotoxicity results in A549 are already available such as those related to AgNP acute exposure [33, 34];
- after short-term exposure (4–24–48 h) at doses ranging from 1 to 100 *μ*g/mL and prolonged (up to 10 days) exposure at low doses ranging from 0.5 to 50 *μ*g/mL.

The cellular biological responses after AgNP treatment included the evaluation of mitochondrial function (using MTT test), membrane integrity and cellular morphology (by calcein-AM/propidium iodide staining), cellular growth, and proliferation (clonogenic tests for sublethal effects).

Silver nitrate (AgNO_3_) has been concomitantly tested, in all experiment types, as positive control for silver toxicity caused by metal ionic form added as soluble salt.

The choice of AgNP size and concentrations was based on previously reports indicating than AgNPs (20 nm) possessed high cytotoxic properties with respect to permeating and damaging cerebral microvascular structures, as compared with larger particles (40 and 80 nm) [29]. Smaller nanoparticles (20 nm) have also been shown to induce higher levels of cellular oxidative damage [26]. Experiments conducted with 20–40 nm AgNPs and using concentrations ranging from 1 to 100 *μ*g/mL to examine the potential hazardous effects of AgNPs with rat primary neuronal cells have found that AgNPs affect voltage-gated sodium and potassium channels, disturb neuronal calcium homeostasis, and reduce dopamine concentration [24–26].

Based on these studies, we pursued to identify the effects not only after AgNP acute exposure but also after continuous exposure to AgNPs (20 nm) at low doses of 0.5, 1, 10, 25, and 50 *μ*g/mL on human CNS cell viability, metabolic activity, membrane integrity, and cell morphology.

## 2. Materials and Methods

### 2.1. Chemicals

All cell culture reagents, culture medium, chemicals, and silver nitrate (AgNO_3_) were purchased from Sigma-Aldrich (Milan, Italy).

### 2.2. Physicochemical Characteristics of Silver Nanoparticles (AgNPs)

AgNPs were supplied by Colorobbia (Colorobbia S.p.A., Vinci, Italy), series PARNASOS NAMA 39 1103 F01 1%. The characteristics of AgNP 1% in water with the appearance of brown colour were 1 g/cm^3^ density, 3 mPa/sec viscosity (25°C), <0.50 PdI, 6.5 pH, and 20 nm nominal hydrodynamic size diameter (20–100 nm size distribution determined by Dynamic light scattering by Malvern Instruments Ltd.).

### 2.3. *In Vitro* Studies

#### 2.3.1. Cell Line and Cell Culture

Human neuroblastoma (SH-SY5Y cell line purchased from ECACC, Sigma-Aldrich, Milan, Italy), human astrocytoma cells (D384 clonal cell line was established from [35]), and human lung epithelial cells (A549 cell line purchased from ECACC, Sigma-Aldrich, Milan, Italy) were used for *in vitro* studies of the AgNP toxicity after short- (4–48 h) and long-term (7–10 days) exposure.

SH-SY5Y cells were cultured in Eagle's minimum essential medium and Ham's F12 (1 : 1) with 15% fetal bovine serum (FBS), 2 mM L-glutamine, 50 IU/mL penicillin, and 50 *μ*g/mL streptomycin.

D384 cells were cultured in Dulbecco's modified Eagle medium (DMEM) supplemented with 10% FBS, 2 mM L-glutamine, 50 IU/mL penicillin, 50 *μ*g/mL streptomycin, and 1% sodium pyruvate. A549 cells were cultured in DMEM supplemented with 10% FBS, 2 mM L-glutamine, 50 IU/mL penicillin, and 50 *μ*g/mL streptomycin. Cells were maintained at 37°C in a humidified atmosphere (95% air/5% CO_2_).

Stock solutions were prepared by dissolving AgNPs in culture medium; cells were exposed to concentrations ranging from 0.5 to 100 *μ*g/mL. For comparison, equivalent amount of AgNO_3_ was tested. Fresh solutions of test materials were prepared shortly before each experiment.

### 2.4. Cytotoxicity Study: Short-Term Exposure (4–48 h)

#### 2.4.1. Mitochondrial Function (MTT Assay) and Membrane Integrity (Calcein-AM/Propidium Iodide Staining)

The cellular viability, membrane integrity, and cell morphology of SH-SY5Y, D384, and A549 cells treated with AgNPs or AgNO_3_ were determined using two colorimetric methodologies: MTT assay enables accurate, straightforward quantification of changes in metabolic activities (i.e., mitochondrial function) and calcein-AM/propidium iodide (PI) staining allows qualitative evaluation on membrane integrity and cell morphology and quantitative evaluation on cell viability (cell live/cell death).

Cells were seeded in 96-well plates at density of 1 × 10^4^ cells/well in complete medium. After 24 h of cell attachment, the cells were exposed to AgNPs or AgNO_3_ at final concentrations between 1 and 100 *μ*g/mL for 4, 24, and 48 h at 37°C.

At the end of the incubation period, the mitochondrial function was assessed by 0.5 mg/mL MTT (3-(4,5-dimethylthiazol-2-yl)-2,5-diphenyltetrazolium bromide) for 3 h at 37°C and was quantified spectrophotometrically at 550 nm in Bio-Rad microplate reader. Data were expressed as a percentage of control (untreated cells).

The membrane integrity and cell morphology were evaluated by the coincubation of the double staining: 2 *μ*M calcein-AM and 2.5 *μ*g/mL PI for 10 min at 37°C. Cells were examined under a Zeiss Axiovert 25 fluorescence microscope combined with digital camera (Canon powershot G8). The fluorescence images were taken using 32x objective lens with an excitation wavelength of 400, 495, and 570 nm, beamsplitter wavelength of 410, 505, and 585 nm, and an emission wavelength of 460, 530, and 610 nm. Viability was expressed as % of cells that retained calcein (green fluorescence) compared to the total cells counted (calcein-positive plus PI-positive (red fluorescence)).

### 2.5. Cytotoxicity Study: Long-Term Exposure (10 Days)

#### 2.5.1. Clonogenic Assay

The procedure for clonogenic assay with the presently used cell types was previously described [36]. Briefly, cells were seeded in six-well plates at density of 300 cells/well for SH-SY5Y cells, 50 cells/well for D384 cells, and 400 cells/well for A549 cells, each well containing 2 mL of cell culture medium. After attachment (about 20 h for SH-SY5Y, 4 h for D384 cells, and 14 h for A549 cells; each time was shorter than the population doubling time 48, 9, and 24 h, resp.) the cells were washed with 2 mL PBS and treated with 2 mL AgNPs (final concentration ranging from 0.5 to 50 *μ*g/mL in cell culture medium) or AgNO_3_, over a time period required to form colonies (about 10 days for SH-SY5Y and A549 cells and 7 days for D384 cells). A colony being defined as at least 50 clones of one cell. At the end of the treatment, the medium was removed and the colonies were fixed, stained with hematoxylin, and then were manually counted for the evaluation of cell survival after AgNP and AgNO_3_ treatments. The minimum size of colony was considered to be 50 cells/colony. The colonies were examined under Zeiss Axiovert 25 microscope combined with a digital camera (Canon powershot G8). Digital photographs were taken from each well using 2.5x objective lens. The number of colonies that arose after treatment was expressed in terms of plating efficiency (PE). PE was calculated by dividing the number of colonies formed by the number of cells plated per 100.

### 2.6. Statistics

Data from short-term exposure were obtained from three independent experiments, each experiment was carried out in six replicates. Data from long-term exposure were obtained from two independent experiments and each experiment was carried out in three replicates. Results are expressed as mean ± SD. Statistical analysis was performed by one-way ANOVA followed by Tukey's test (for each time point). A value of *P* < 0.05 was considered statistically significant.

Cytotoxicity data by MTT was fitted to an exponential growth model in order to calculate the 50% effective concentration (EC_50_). This analysis was performed using the REGTOX-EV7.xls curve fitting add-in macro for Microsoft Excel (http://www.normalesup.org/~vindimian/macro/REGTOX_EV7.0.6.xls).

## 3. Results

### 3.1. Cytotoxic Activity of AgNPs Compared to AgNO_3_ in Human Nervous (SH-SY5Y and D384 Cell Lines) and Pulmonary Cells (A549 Cell Line)

*In vitro* cytotoxicity due to the short (4–24–48 h) and prolonged (7 or 10 days) exposure of SH-SY5Y, D384, and A549 cells to increasing concentrations of AgNPs (from 0.5 to 100 *μ*g/mL) and AgNO_3_ is reported and compared. Mitochondrial function, membrane integrity, and cell morphology were considered as endpoints of acute exposure, while the capacity to form colonies was considered as endpoint of chronic exposure.

### 3.2. Cytotoxicity Results after Short-Term Exposure (4–48 h)

#### 3.2.1. Mitochondrial Function. MTT Assay

**Figure 1:**
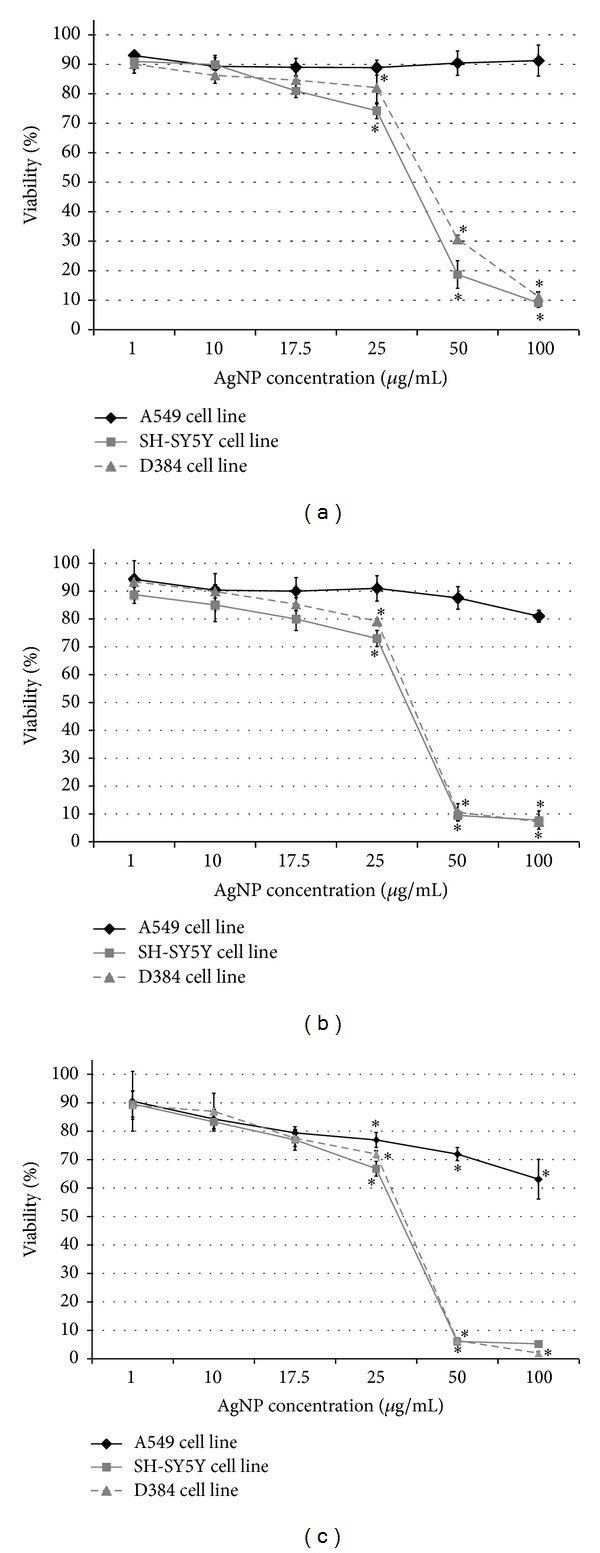
Mitochondrial function evaluation by MTT assay in SH-SY5Y, D384, and 549 cell lines. Evaluation of mitochondrial function in SH-SY5Y, D384, and A549 cell lines after 4 h (a), 24 h (b), and 48 h (c) exposure to increasing concentrations (from 1 to 100 *μ*g/mL) of AgNPs showed dose- and time-dependent changes in mitochondrial metabolism on both cerebral cell lines starting at the dose of 25 *μ*g/mL and already after 4 h exposure. Pulmonary cells (A549) were less susceptible to AgNP exposure, showing a reduction of cell viability after 48 h exposure at the higher doses of AgNPs. *Different from control in each cell line *P* < 0.05, Statistical analysis by ANOVA followed by Tukey's test. Error bars indicate S.D.

Data of mitochondrial function, evaluated by MTT after 4, 24, and 48 h of exposure to increasing concentrations of AgNPs (1–100 *μ*g/mL) and expressed as percentage of the viability of control, are presented in Figure 1. AgNPs induced dose-dependent cytotoxic effects on both SH-SY5Y and D384 cells; there was a strong cell viability decrease already after 4 h exposure (Figure 1(a)); loss of cell viability was about 25–85% at doses ranging from 25 to 100 *μ*g/mL, and no effect was observed from 1 to 17.5 *μ*g/mL. The cytotoxic effect of AgNPs for both cerebral cell types was shown to be more pronounced after 24 and 48 h exposure as indicated by about 100% reduction of cell viability at the highest tested doses (50–100 *μ*g/mL) (Figures 1(b) and 1(c)). A549 cells were less susceptible than cerebral cells, showing about 35% cell viability reduction after 48 h exposure to the highest tested dose of 100 *μ*g/mL AgNPs (Figure 1(c)).

In addition, MTT data were used to calculate EC_50_ (50% effective concentration) values and were used to compare the toxicity rank of AgNPs on SH-SY5Y, D384, and A549 cell lines. As illustrated in Table 1, both the EC_50s_ of SH-SY5Y and D384 were observed to be dependent on the dose used and time period of exposure, while the EC_50_ of A549 was significantly greater than the highest dose of AgNP tested, indicating that A549 cells were less susceptible to AgNP treatment compared to SHSY5Y and D384 cells.

**Figure 2:**
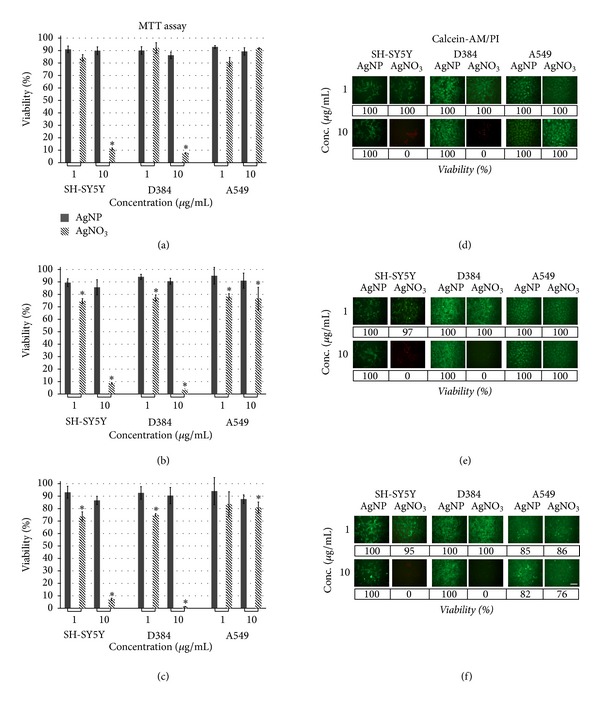
Data comparisons between AgNPs and AgNO_3_ in SH-SY5Y, D384 and A549 cell lines. Panels (a), (b), and (c) show mitochondrial function evaluation by MTT assay after 4 h, 24 h and 48 h exposure to 1 and 10 *μ*g/ml of AgNPs and AgNO_3_. *Different from control in each cell line *P* < 0.05, statistical analysis by ANOVA followed by Tukey's test. Error bars indicate S.D. Panels (d), (e), and (f) show membrane integrity by calcein-AM/Propidium Iodide staining after 4 h, 24 h and 48 h exposure to 1 and 10 *μ*g/ml of AgNPs and AgNO_3_; scale bar 100 *μ*m. AgNO_3_ produced a more pronounced cytotoxic effect to that caused by comparable amount of AgNPs on cerebral cell lines. In A549 cells, similar low cytotoxic profile was observed after both treatment types (AgNPs and AgNO_3_).

*AgNP versus AgNO*_*3*_ *Comparison.* Using AgNO_3_ at 1 and 10 *μ*g/mL, the noncytotoxic doses for AgNPs, a strong toxic effect was observed especially at 10 *μ*g/mL in both cerebral cell lines (SH-SY5Y and D384 cells) and for each considered time point (i.e., 4, 24, and 48 h), while in A549, the AgNO_3_ effect was less pronounced (Figures 2(a), 2(b), and 2(c)).

Specifically, up to about 18 *μ*g/mL AgNPs did not produce any cytotoxic effect within 48 h in all considered cell types differently from AgNO_3_ that showed to be very toxic (especially at 10 *μ*g/mL) in the cerebral cell lines and weakly effective in A549.

#### 3.2.2. Membrane Integrity: Calcein-AM/PI Staining

**Figure 3:**
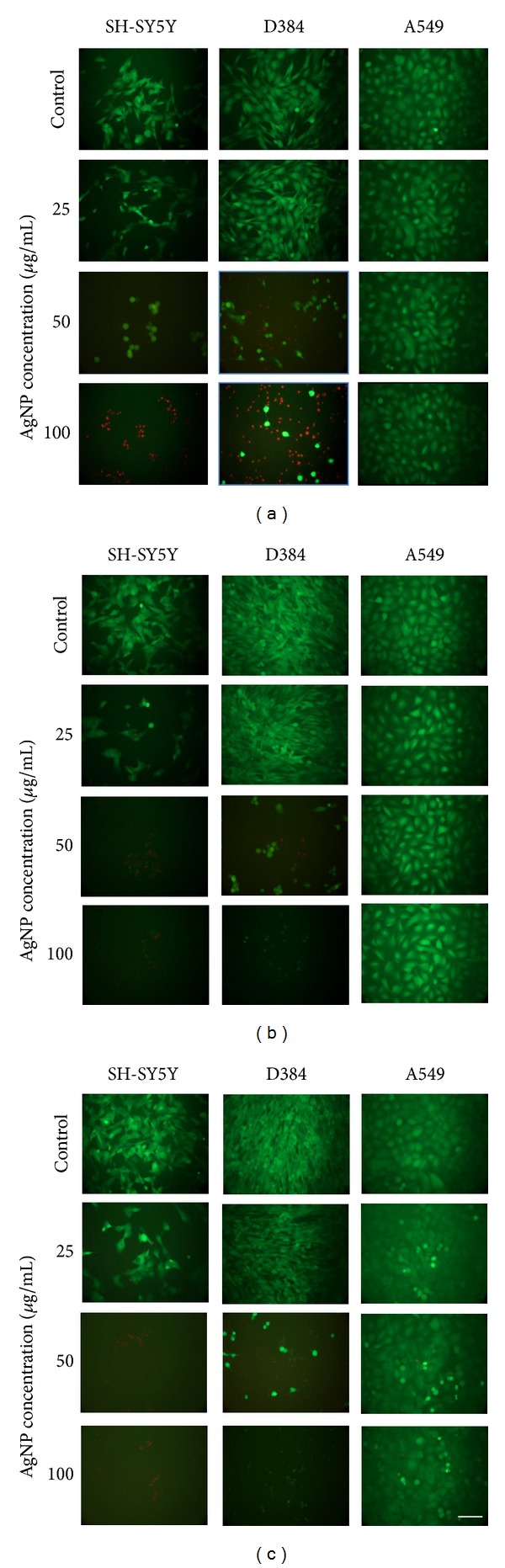
Membrane integrity evaluation. Representative images of randomly selected microscopic fields of cells (SH-SY5Y, D384, or A549) stained with calcein-AM/propidium iodide after 4 (a), 24 (b), and 48 (c) h exposure to increasing concentrations (25–100 *μ*g/mL) of AgNPs. Green fluorescence patterns and the cellular morphology were similar to the controls. A strong decrease in cell viability for both cerebral cell lines was observed as evidenced by the presence of numerous red coloured cells (indicating damage to the cell membrane) at the highest doses (50–100 *μ*g/mL). After 24 and 48 h, the cytotoxic effect of AgNPs was exacerbated. SH-SY5Y cells were the most sensitive towards AgNP since the cell loss was evident at the dose of 25 *μ*g/mL, whereas pulmonary cells (A549) were less susceptible to AgNP exposure compared to cerebral cell types, showing a slight decrease of cell viability after 48 h exposure only (scale bar is 100 *μ*m).

Membrane integrity and cell morphology were evaluated by calcein-AM/PI staining after 4, 24, and 48 h exposure to increasing concentrations of AgNPs (1–100 *μ*g/mL).  Figure 3 describes a panel of representative and randomly selected microscopic fields of SH-SY5Y, D384, and A549 cells treated with AgNPs. Effect on cell viability was also confirmed using calcein-AM/PI double staining for all cell types.

The green fluorescence of SH-SY5Y and D384 cells after 4 h exposure was uniformly diffused in cell cytoplasm (indicating the maintained membrane integrity) at doses ranging from 1 to 25 *μ*g/mL and cell morphology was not altered with respect to control (1–10 *μ*g/mL data are shown in the Supplementary additional file 1, available online at http://dx.doi.org/10.1155/2014/259765; it reports membrane integrity evaluation by Calcein-AM/PI staining of SH-SY5Y, D384, or A549 cells after 4, 24, and 48 h exposure to increasing concentrations (1–100 *μ*g/ml) of AgNPs.  Figure 3 shows 25–100 *μ*g/mL), while a strong decrease in cell viability for both cell lines was observed as evidenced by the presence of numerous red coloured cells (indicating damage to the cell membrane) at the highest doses (50–100 *μ*g/mL) (Figure 3).

After 24 and 48 h, the cytotoxic effect of AgNP was exacerbated as even shown by semiquantitative analysis (Table 2) of selected microscopic fields, in terms of cell counts and expressed as percentage of live cells (green fluorescence). SH-SY5Y cells were more sensitive towards AgNPs since the cell loss was evident at the dose of 25 *μ*g/mL (Figure 3; Table 2).

Fluorescence images of A549 cells (Figure 3) showed uniformly diffused green fluorescence and normal cell morphology for all treatment concentrations (1–100 *μ*g/mL) and at each time point (4, 24, and 48 h) when compared to control. Semiquantitative analysis on A549 cells with increasing concentrations of AgNPs (1–100 *μ*g/mL) showed a slight decrease of cell viability (about 15–20%) after 48 h exposure only (Table 2).

*AgNP versus AgNO*_*3*_ *Comparison*. Cytotoxicity induced by AgNO_3_ and evaluated with calcein-AM/PI still shows to be pronounced at 1 and 10 *μ*g/mL (noncytotoxic doses for AgNPs) for both SH-SY5Y and D384 cells and at each considered time point (i.e., 4, 24, and 48 h), while, in A549, both AgNO_3_ and AgNPs produced similar cytotoxic effect for both 1 and 10 *μ*g/mL doses (Figures 2(d), 2(e), and 2(f)).

### 3.3. Cytotoxicity Results after Long-Term Exposure (10 Days)

#### 3.3.1. Clonogenic Assay

**Figure 4:**
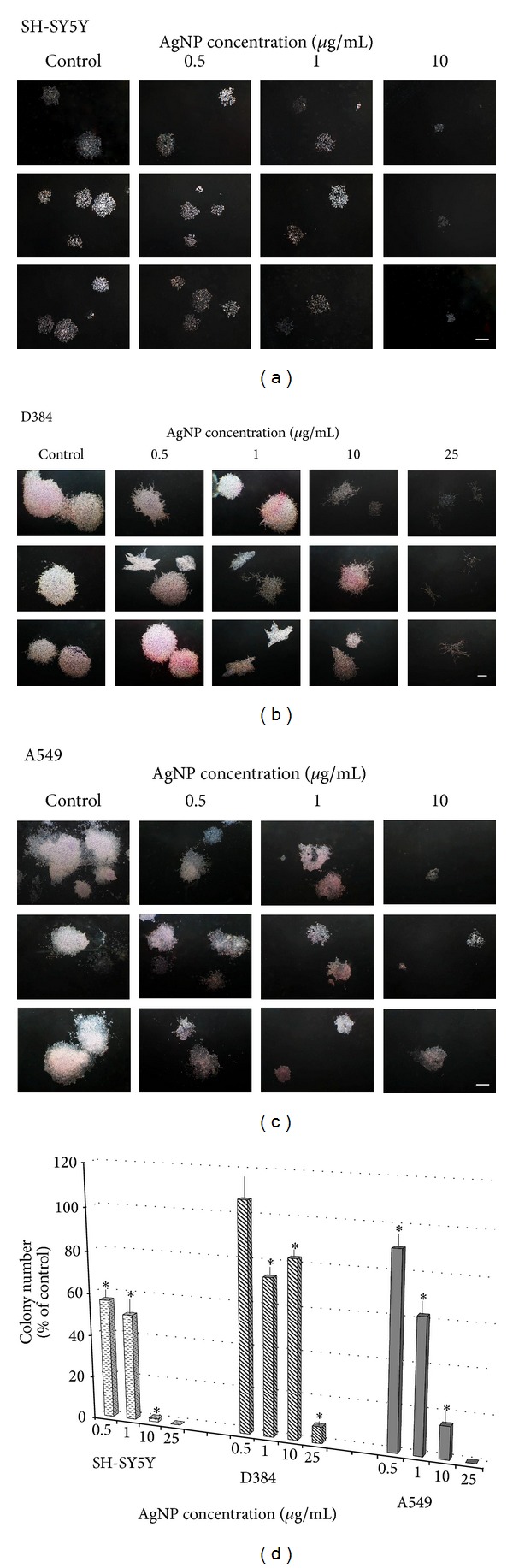
Clonogenic assay up to 10-day exposure. The images of randomly selected microscopic fields show the colonies of SH-SY5Y (a), D384 (b), and A549 (c) cells formed after 7 or 10 consecutive days of exposure to increasing concentration of AgNPs (0.5–25 *μ*g/mL). The colonies of all cell types treated with AgNPs showed dose-dependent reductions on size, colony number, and changes in colony morphology compared to each respective control (scale bar is 600 *μ*m). Panel (d) shows a semiquantitative analysis in SH-SY5Y, D384, and A549 cell lines: histograms display the number of colonies formed after 7 or 10 consecutive days of exposure to increasing concentration of AgNPs (0.5–25 *μ*g/mL). Dose-dependent inhibition of colony formation from all cell models was observed, in particular cell sensitivity was SH-SY5Y > A549 > D384. Decrease in SH-SY5Y and D384 colony number was 50% and 25%, respectively, at 1 *μ*g/mL, and exposure to even lower AgNP dose, such as 0.5 *μ*g/mL, was significantly effective towards SH-SY5Y and pulmonary cells. Data are expressed as percentage of respective control colonies. Error bars are ±S.D. *Significant decrease of colony number: different from control (*P* < 0.05), statistical analysis by ANOVA followed by Tukey's test.

To determine whether the prolonged exposure (up to 10 days) to increasing AgNP concentrations (0.5–50 *μ*g/mL) might cause adverse effects, the proliferation ability and colony forming capacity of SH-SY5Y, D384, and A549 cells were evaluated.  Figure 4 shows representative images of randomly selected microscopic fields of the different cell types.

Colonies of SH-SY5Y and D384 treated with increasing concentrations of AgNPs (0.5–25 *μ*g/mL) and colonies of A549 treated with AgNPs (0.5–50 *μ*g/mL) showed dose-dependent reductions on size and colony number, as well as changes in colony morphology compared to each respective control (Figures 4(a), 4(b), and 4(c)). Semiquantitative analysis showed a strong reduction (about 90%) of colony number of D384 at the highest treatment dose of 25 *μ*g/mL, and SH-SY5Y cells were totally inhibited (Figure 4(d)). AgNP effect was more pronounced on SH-SY5Y compared to D384: 50% versus 25% decrease in colony number for SH-SY5Y and D384, respectively, at 1 *μ*g/mL, and 45% decrease was already observed in SH-SY5Y at the lowest tested dose of 0.5 *μ*g/mL.

Different from short-term exposure data, A549 cells showed to be sensitive towards AgNP exposure already after applying the lowest dose of 0.5 *μ*g/mL. Moreover, 75% decrease in colony number was observed at the dose of 10 AgNP *μ*g/mL, and total inhibition was observed at doses from 25 to 50 *μ*g/mL (Figure 4).

**Figure 5:**
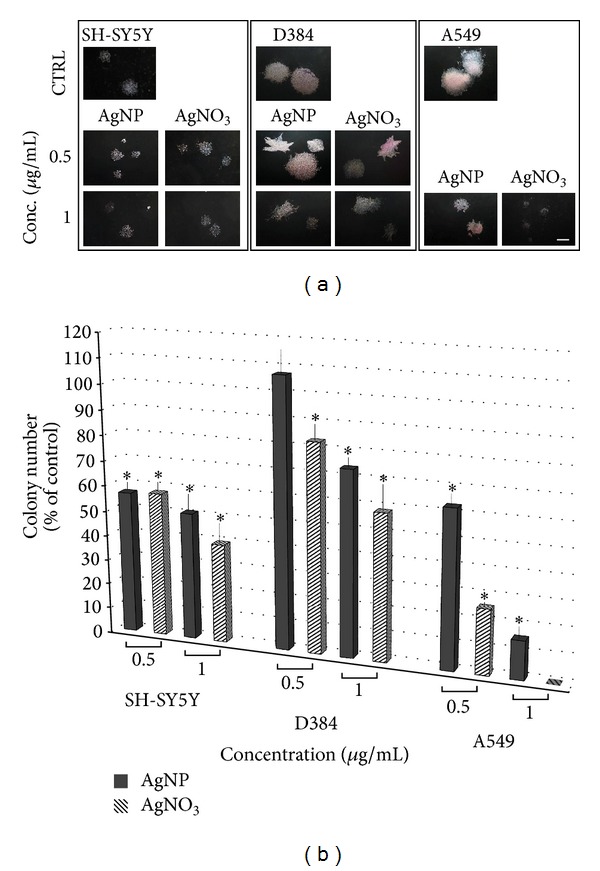
Data comparison between AgNPs and AgNO_3_ in SH-SY5Y, D384, and A549 cells for clonogenic assay. Panel (a) shows images of selected microscopic fields from control and treated cells with AgNPs or AgNO_3_ at 0.5 and 1 *μ*g/mL for up to 10 days (scale bar is 600 *μ*m). Panel (b) shows the respective semiquantitative analysis for the colony number of SH-SY5Y, D384, and A549 after AgNPs or AgNO_3_ treatment. AgNO_3_ induced a more pronounced effect on growth and cell proliferation than AgNP treatment regardless of cell model type. *Different from control in cell line *P* < 0.05, statistical analysis by ANOVA followed by Tukey's test.

*AgNP versus AgNO*_*3*_ *Comparison*. Data comparison between AgNP and AgNO_3_ indicated that the latter caused more pronounced effects than those produced by AgNP towards all cell types, that is, cerebral and pulmonary (Figure 5). In particular, in A549 cells, 1 *μ*g/mL of AgNO_3_ treatment produced a total blockage of the colony formation (i.e., inhibition of cell proliferative activity), while similar amount of AgNP caused 85% reduction (Figure 5(b)).

## 4. Discussion

The present study provides the first cytotoxic evidence that exposure of human cerebral SH-SY5Y and D384 cell lines to AgNPs causes cytotoxic effects not only after short-term exposure (4–48 h) altering mitochondrial metabolism, membrane integrity, and morphology but also after long-term exposure (up to 10 days), at particularly low doses, compromising growth and cell proliferation.

The major results obtained after short-term exposure (4–48 h) indicate that:

- AgNP treatment produced dose- and time-dependent neurotoxic effects, as indicated by changes in mitochondrial metabolism and damage to the cell membrane, on cerebral cell lines (SH-SY5Y and D384) starting at the dose of 25 *μ*g/mL and after 4 h exposure. EC_50_ values were 40.7 ± 2.0 and 49.5 ± 2.1 *μ*g/mL for SH-SY5Y and D384, respectively, after 4 h exposure. Pulmonary cells (A549) were less vulnerable to AgNP exposure compared to cerebral cell types;
- low doses (up to about 18 *μ*g/mL) of AgNPs did not produce any cytotoxic effect within 48 h;
- the magnitude of effects caused by AgNO_3_ was more pronounced compared to that produced by comparable amount of AgNPs on cerebral cell lines: at 10 *μ*g/mL AgNPs did not produce cytotoxic effect in all cell types considered differently from AgNO_3_ that showed to be very toxic. In A549 cells analogous low cytotoxic profile was observed after both treatment types (AgNPs and AgNO_3_).

Data achieved after long-term exposure (up to 10 days) revealed that:

- extremely low doses (≤1 *μ*g/mL) of AgNPs were critical for cell viability since they were able to compromise the proliferative capacity. The effect was evident regardless of cell model type; cell sensitivity was SH-SY5Y > A549 > D384. Decrease in SH-SY5Y and D384 colony number was 50% and 25%, respectively, at 1 *μ*g/mL, and exposure to even lower AgNP dose, such as 0.5 *μ*g/mL, was significantly effective towards SH-SY5Y and pulmonary cells;
- different from what was observed in short-term exposure experiments, A549 cells were very sensitive towards a prolonged exposure to AgNPs: 10 *μ*g/mL caused 75% colony number decrease and ≥25 *μ*g/mL suppressed cell growth;
- AgNO_3_ induced a more pronounced effect on growth and cell proliferation than AgNP treatment.

Regarding the AgNP short-term exposure (4–48 h), we used viability assays (namely, MTT test and Calcein-AM/PI), vital steps in toxicology that explain the cellular response to a toxicant. They give information on cell death, survival, and metabolic activities. The mitochondrial damage is indicated by the reduced dehydrogenase activity as measured by the reduction of tetrazolium dye (MTT), which takes place at the ubiquinone and cytochrome b and c sites of the mitochondrial electron transport chain [37]. Membrane integrity is indicated by intracellular esterases activity, while cells with cell membrane damaged are detected by propidium iodide intercalating between the bases of nucleic acids. Simultaneously both live and death cells are marked; the damage in the cell membrane indicates that more cell population is possibly dying by either necrotic or apoptotic pathway.

Our data using these endpoints clearly indicate that no alteration can be detected up to 18 *μ*g/mL in all cell types (cerebral and pulmonary). With respect to the cerebral cell lines, these results are in accordance with data of Luther et al. [23] showing that acute exposure (4 h) of primary astrocytes to AgNPs (1–100 *μ*M corresponding to about 1.78–10.78 *μ*g/mL) did not compromise cell viability despite leading to a concentration-dependent increase in the specific cellular silver content to up to 46 nmol/mg protein. During a subsequent incubation of the cells in AgNP-free medium, the cellular silver content of AgNP-treated astrocytes remained almost constant for up to 7 days, but the cellular presence of AgNP did neither induce any delayed cell toxicity nor compromise basal metabolism. Nevertheless, AgNP-treated astrocytes strongly upregulated the expression of metallothioneins which (as suggested by the authors) could have helped prevent silver-mediated toxicity induced by possibly AgNP derived silver ions.

In acute (24 h) experiments, using mixed rat primary neuronal cell cultures (astrocytes and neurons) exposed to AgNP 5–10 *μ*g/mL, a significant cytotoxic effect was observed at 10 *μ*g/mL as well as grossly morphological disorganization of the astrocytes but not neurons, and this seems consistently with the finding that AgNPs were mainly taken up by astrocytes and not by neurons. At higher concentration such as 20 *μ*g/mL AgNPs, both cell types exhibited an equally affected morphology [26].

Other experiments conducted with 20–40 nm AgNPs (1–100 *μ*g/mL) to examine the potential hazardous effects of AgNPs with rat primary neuronal cells demonstrated that AgNPs inhibited neuronal sodium and potassium currents at 10 *μ*g/mL, disturbed neuronal calcium homeostasis at 5 *μ*g/mL, and reduced dopamine concentration at 50 *μ*g/mL [24, 25]. On the other hand, up to now, effects due to acute exposure to a noncytotoxic AgNP doses, such as <0.5 *μ*g/mL, were observed only in human epathoma cells (HepG2) in which induced gene expression associated with the cycle progression and apoptosis was reported [38].

The present study clearly demonstrated that cerebral cells (SH-SY5Y and D384) were more vulnerable (effects at concentrations > 10 *μ*g/mL AgNPs) than A549 cells in all the short-term cytotoxicity assays; the toxic effects in A549 are evident only 48 h after exposure to the highest dosages (50–100 *μ*g/mL). Indeed, a recent study indicated no toxicity for AgNPs (up to 10 *μ*g/mL of silver) on A549 cells although a high cellular uptake was demonstrated. This lack of toxicity was suggested to be most likely due to low intracellular release of silver ions from AgNPs within short-time periods (4–24 h) [39]. Silver ions seemed to be toxic via extracellular mechanisms causing cell membrane damage; hypothesis based on the evidence that silver ions dissolved from AgNO_3_ (with a relatively low cellular penetration capacity) induced much more cell death in terms of loss in cell membrane integrity compared to the exposure to AgNP that did not cause any effect despite its high cellular uptake and high dosage of silver.

The present results obtained after long-term treatment with AgNP suggest that continuous exposure to low AgNP doses (≤1 *μ*g/mL) severely affected proliferation of all cell types (SH-SY5Y, D384, and A549).

We used clonogenic test, a noncolorimetric assay, to determine the proliferation ability of all cell types upon long-term exposure to AgNPs. Clonogenic assay is a cell survival assay using the ability of cells to form colonies when seeded at very low cellular concentrations; it identifies the cells that are destined to die or survive. Studies using the clonogenic assay to evaluate cell survival after exposure to nanomaterials are very limited. However, it has been found that this assay was suitable for testing the toxicity of carbon nanotubes [40, 41].

Recently, concentration-dependent effects on the proliferation ability of RBE4 cells (microvascular endothelial cells important for proper function of BBB) exposed to different-sized (10, 50, and 100 nm) citrate-coated AgNPs for up to 5 days were observed; the colony number was reduced after exposure to 0.01 and 0.1 *μ*g/mL AgNPs (10 nm) and was totally suppressed at 5 *μ*g/mL [42].

Few data on cultured neurons [27] other than ours indicate that AgNP induces toxicity in cerebral cells at remarkably low doses of 0.5–1 *μ*g/mL in conditions of prolonged AgNP presence. In particular, cultured rat cortical neurons exposed to 1 *μ*g/mL of AgNPs for 2-3 days exhibited compromised cell morphological integrity, degradation of synaptic proteins, and degeneration of cytoskeletal proteins (beta-tubulin and F-actin) [27]. Our results indicate that a longer period of exposure (up to 10 days) to AgNP concentrations even below 1 *μ*g/mL (i.e., 0.5 *μ*g/mL) led to a reduced size and colony number as well as changes in colony morphology in SH-SY5Y and pulmonary cell lines compared to untreated cells. SH-SY5Y cells were apparently more susceptible against AgNP treatment when compared with D384 cells (toxic effect at 0.5 *μ*g/mL for SH-SY5Y and 1 *μ*g/mL for D384). This finding is of special interest due to, on one side, the recognized particular role of astrocytes in several neurodegenerative diseases; being more likely, the cell type initially affected during pathogenesis [43]. On the other hand, astrocytes have a variety of important functions such as supplying of metabolic nutrients to neurons and protecting the brain against oxidative stress and metal toxicity [44–47]. Astrocytes communicate with neurons to enable synapse formation, synaptic transmission, plasticity, and synaptic homeostasis [48, 49]. The high vulnerability of neurons other than astrocytes observed after long-term exposure to low AgNP doses, as demonstrated here, might thus have fundamental consequences on the proper function of neural networks. In addition, several studies have also reported that the rate of nanoparticle translocation into the brain can be significantly increased under certain pathological conditions, such as infection, meningitis, and systemic inflammation [10, 14].

The *in vivo* relevance of these cell culture data should therefore be addressed to explore the CNS effects in *in vivo* situation. So far, the few *in vivo* rodent studies have mostly used high level exposure to AgNPs indicating AgNP-induced significant toxicity to a variety of organs including lung, liver, and brain (see review of [50]) with brain appearing as the most sensitive organ. Increased Ag concentrations in the rat brain and olfactory region (about 1.4 and 1.9 ng/g wet weight, resp.) immediately and (about 1.2 and 3.1 ng/g, resp.) one day after 6 h inhalation exposure to 15 nm AgNPs (cumulative dose of 7.2 *μ*g) were reported [51]. In rats subcutaneously injected with AgNPs (<100 nm) at 62.8 mg/kg bw, silver crossed the blood-brain barrier and accumulated in the brain (starting at 2 weeks after exposure with levels of 39 ± 18 ng/brain) along with other organs. Significantly higher Ag tissue content, than in the control group, was observed from 4 to 24 weeks (165 ± 71 and 362 ± 120 ng/brain, resp.) after exposure and increased incidence of astrocyte swelling and neuronal degeneration was reported from 2 to 24 weeks after exposure due to the accumulation of AgNPs [12].

Altogether, these *in vivo* studies (relevant although with high Ag dosage) along with recent *in vitro* results, including ours (especially from prolonged exposure to AgNPs), encourage additional research and investigation addressing chronic low-dose AgNP exposure that would be useful to translate a realistic human chronic exposure scenario.

Despite several recent *in vitro* and *in vivo* publications on AgNP toxicity, the mechanism of AgNP toxicity remains unclear. The most critical question is whether AgNP toxicity is mechanistically unique to nanoparticulate silver or it is the results of the release of silver ions (Ag^+^), a well-known molecular toxicant, or it is the combination of both.

Actually we did not measure the Ag^+^ cellular uptake and release from NPs. The latter seems to depend on a variety of factors, such as particle size, the medium used to disperse the NPs, the temperature, the particles crystallinity, and the surface functionalization [52, 53]. Several studies suggest that the mechanism of AgNP toxicity is largely explained by Ag ions (Ag^+^). For example, lack of toxicity was observed when Ag^+^ was complexed by a thiol ligand [54–58] or when AgNP was tested under strictly anaerobic conditions that precluded Ag(0) oxidation and Ag^+^ release [59]. Genetic analysis also evidenced that AgNP toxicity was mediated by ionic silver release [60]. Ag^+^ may be released into solution or may be sorbed by the AgNPs and delivered locally at high doses to the cell (i.e., the Trojan horse effect) [61, 62]. Other studies suggest that ion release does not explain all toxicity, and some support a role for generation of reactive oxygen species (ROS), which might occur at the surface of AgNPs [17, 63–66] but is not expected to result from silver ion dissolution alone. One study reported that cysteine, a strong Ag^+^ ligand, only partially rescued AgNP toxicity [38], while others found that AgNP cytotoxicity was independent of Ag^+^ concentration and resulted primarily from oxidative stress [65, 67].

## 5. Conclusion

This study demonstrates cytotoxic effects of AgNPs. The cerebral cell lines are more susceptible than pulmonary ones after acute exposure, while prolonged exposure to low AgNP doses significantly compromises the proliferative capacity of all cell model types.

The relevance of these results is based on the fact that human lung epithelial cells (A549) are representative for common route of human AgNP exposure (e.g., inhalation) and AgNPs are able to translocate from the site of entry to several secondary organs including the CNS.

These *in vitro* approaches (several endpoints and short- and long-term exposure) allow high throughput, replicates, and parallel complementary tests, which can explain the molecular response of cells caused by AgNP exposure and help to design focused *in vivo* realistic studies.

## Supplementary Material

Supplementary Figure: Membrane integrity evaluation after 4-48 h exposure to increasing concentrations of AgNP (1-100 *μ*g/ml). Representative images of randomly selected microscopic fields of cells (SH-SY5Y, D384, or A549) stained with calcein-AM/PI after 4 (A), 24 (B) and 48 (C) h exposure to increasing concentrations (1-100 *μ*g/ml) of AgNPs. Green fluorescence patterns of both cerebral cell lines were similar to the controls at doses ranging 1 to 25 *μ*g/ml at time points (4, 24 and 48 h). A strong decrease in cell viability was observed as evidenced by the presence of numerous red coloured cells (indicating damage to the cell membrane) at the highest doses (50-100 *μ*g/ml). After 24 and 48 h, the cytotoxic effect of AgNPs was exacerbated. Fluorescence images of A549 cells (A, B, C) showed uniformly diffused green fluorescence, and normal cell morphology for all treatment concentrations (1-100 *μ*g/ml), was observed a slight decrease of cell viability after 48 h exposure only (scale bar 100 *μ*m).

## Figures and Tables

**Table 1:** EC_50_ after 4, 24, and 48 h exposure to AgNPs (1–100 *μ*g/mL). Effective concentrations causing 50% (EC_50_ values, *μ*g/mL) loss of cell viability (evaluated by MTT test) for SH-SY5Y, D384, and A549 cell lines following exposure to increasing concentrations of AgNP (0–100 *μ*g/mL).

Cell type	EC_50_ (*μ*g/mL)		
	4 h exposure	24 h exposure	48 h exposure
SH-SY5Y	40.71 ± 2.00	30.73 ± 3.20	28.38 ± 3.00
D384	49.49 ± 2.10	33.41 ± 4.00	30.21 ± 3.40
A549	No cytotoxic effects	No cytotoxic effects	Cytotoxic effects starting at >100 *μ*g/mL (about 65% viability)

**Table 2:** Semiquantitative cell live analysis after 4–48 h exposure to increasing concentrations of AgNPs (1–100 *μ*g/mL). Semiquantitative analysis of selected microscopic fields of SH-SY5Y, D384, and A549 cells after 4, 24, and 48 h exposure to increasing concentrations of AgNPs (1–100 *μ*g/mL), in terms of cell counts and expressed as percentage of live cells (mean ± S.D).

AgNP (*μ*g/mL)	Live cells (%)		
	4 h exposure	24 h exposure	48 h exposure
SH-SY5Y cells			
1	100	100	100
10	100	100	100
25	100	35.5 ± 9.46*	21 ± 5.69*
50	0*	0*	0*
100	0*	0*	0*

D384 cells			
1	100	100	100
10	100	100	100
25	100	100	100
50	59.67 ± 12.40*	41.67 ± 5.11*	6.17 ± 3.20*
100	4.00 ± 6.20*	0*	0*

A549 cells			
1	100	100	84.66 ± 4.08
10	100	100	82.00 ± 2.45
25	100	100	81.66 ± 2.87
50	100	100	79.50 ± 2.88
100	100	100	80.83 ± 2.40

Mean of 6 images for each cell type and each dose of AgNPs. *Statistical analysis by ANOVA with Tukey's test compared to its control (*P* < 0.05).
