# Supplementary material for: Assessment of Cellular Responses after Short- and Long-Term Exposure to Silver Nanoparticles in Human Neuroblastoma (SH-SY5Y) and Astrocytoma (D384) Cells
Source: ScientificWorldJournal. 2014 Feb 13;2014:259765. doi: 10.1155/2014/259765 (PMC3947692; doi:10.1155/2014/259765)
Supplement: Supplementary file 1 — Supplementary Figure: Membrane integrity evaluation after 4-48 h exposure to increasing concentrations of AgNP (1-100 μg/ml). Representative images of randomly selected microscopic fields of cells (SH-SY5Y, D384, or A549) stained with calcein-AM/PI after 4 (A), 24 (B) and 48 (C) h exposure to increasing concentrations (1-100 μg/ml) of AgNPs. Green fluorescence patterns of both cerebral cell lines were similar to the controls at doses ranging 1 to 25 μg/ml at time points (4, 24 and 48 h). A strong decrease in cell viability was observed as evidenced by the presence of numerous red coloured cells (indicating damage to the cell membrane) at the highest doses (50-100 μg/ml). After 24 and 48 h, the cytotoxic effect of AgNPs was exacerbated. Fluorescence images of A549 cells (A, B, C) showed uniformly diffused green fluorescence, and normal cell morphology for all treatment concentrations (1-100 μg/ml), was observed a slight decrease of cell viability after 48 h exposure only (scale bar 100 μm). [file 259765.f1.pdf]

Supplementary Materials
Figure 3 suppl data
Figure 3 Supplement data

Membrane integrity evaluation after 4-48 h exposure to increasing concentrations of AgNP (1-100 µg/ml). Representative images of randomly selected microscopic fields of cells (SH-SY5Y, D384, or A549) stained with calcein-AM/PI after 4 (A), 24 (B) and 48 (C) h exposure to increasing concentrations (1-100 µg/ml) of AgNPs. Green fluorescence patterns of both cerebral cell lines were similar to the controls at doses ranging 1 to 25 µg/ml at time points (4, 24 and 48 h). A strong decrease in cell viability was observed as evidenced by the presence of numerous red coloured cells (indicating damage to the cell membrane) at the highest doses (50-100 µg/ml). After 24 and 48 h, the cytotoxic effect of AgNPs was exacerbated. Fluorescence images of A549 cells (A, B, C) showed uniformly diffused green fluorescence, and normal cell morphology for all treatment concentrations (1-100 µg/ml), was observed a slight decrease of cell viability after 48 h exposure only (scale bar 100 µm).

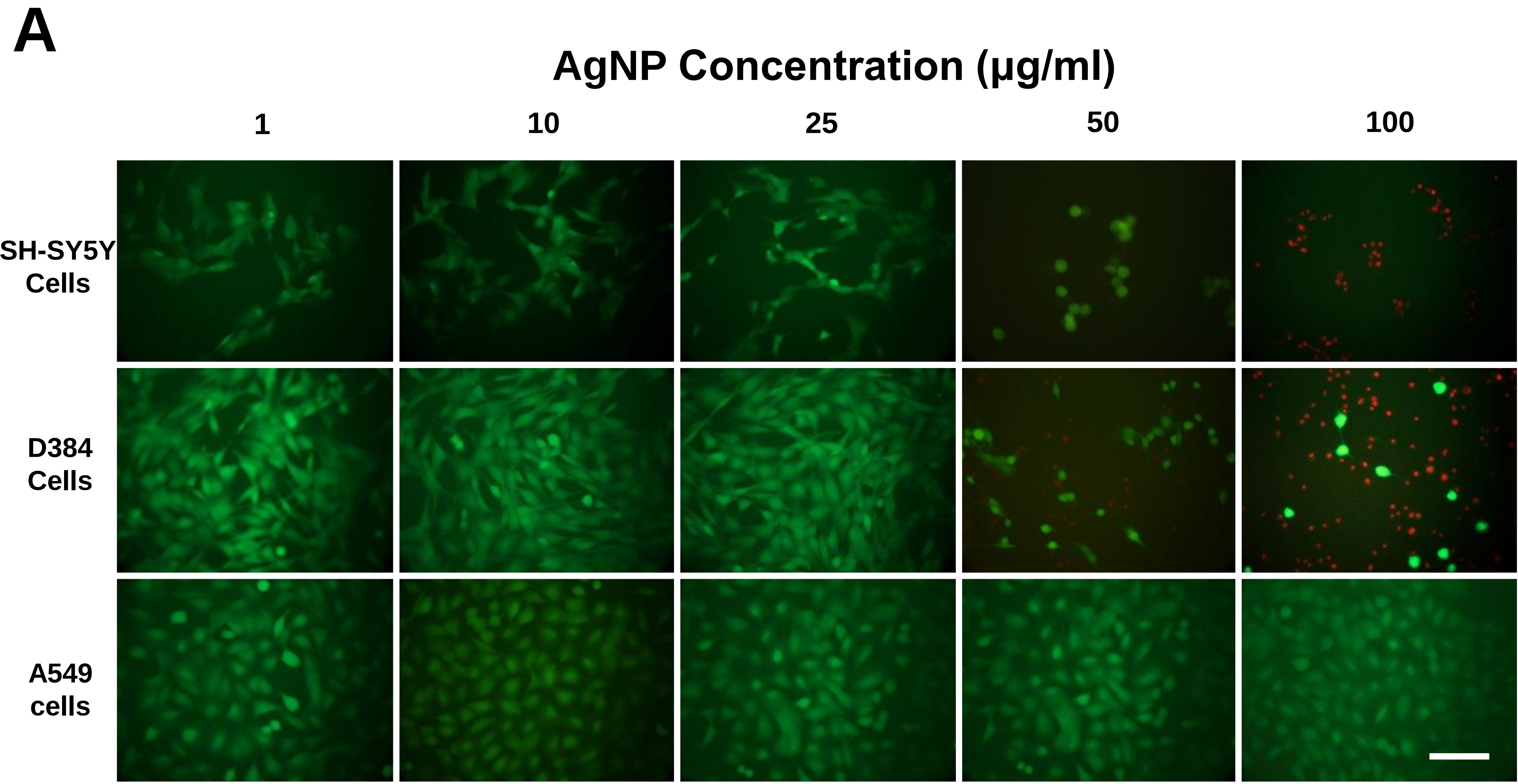
A
AgNP Concentration (μg/ml)
1
10
25
50
100
SH-SY5Y Cells
D384 Cells
A549 cells

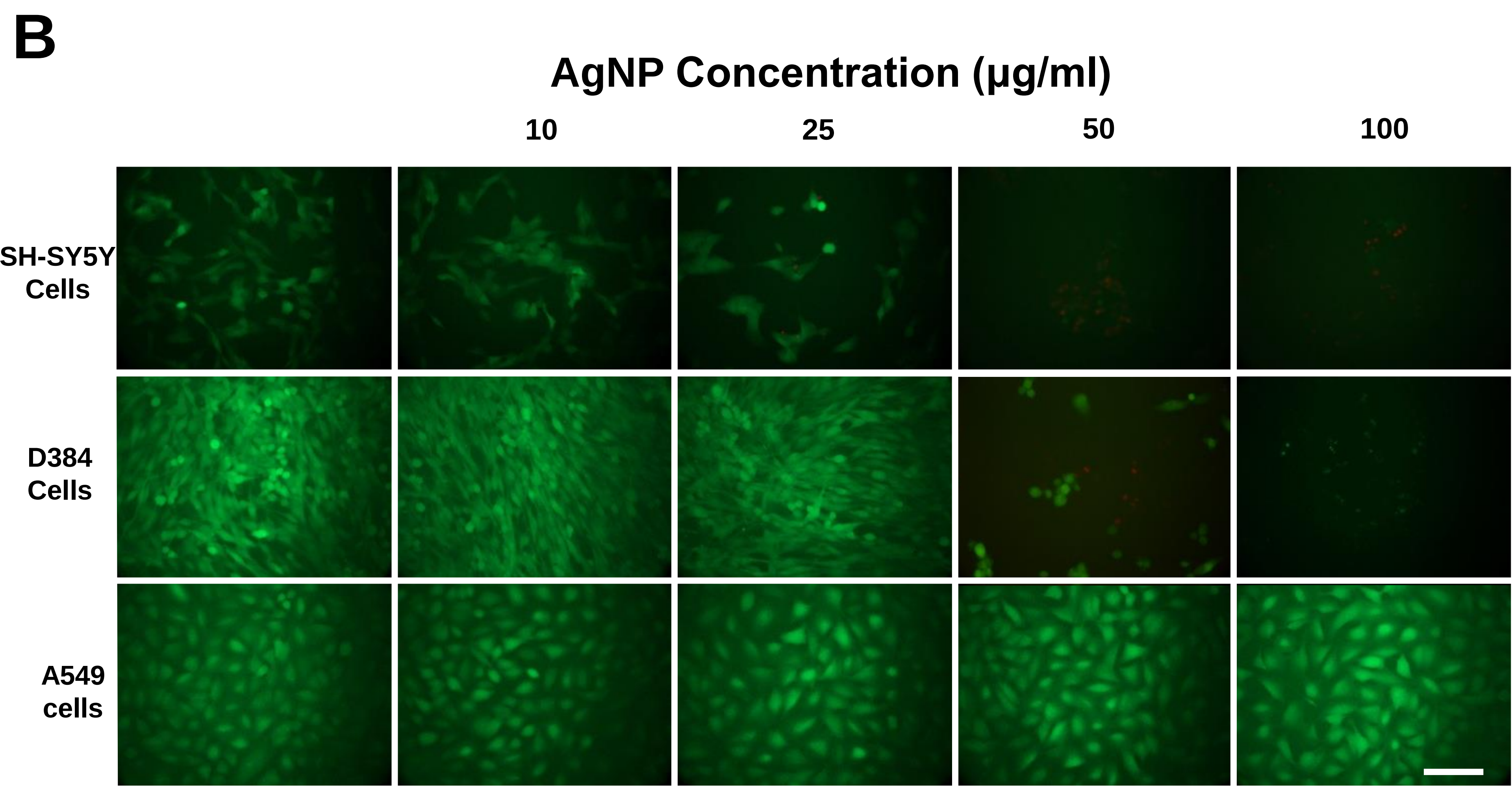
B
AgNP Concentration (µg/ml)
10
25
50
100
SH-SY5Y Cells
D384 Cells
A549 cells

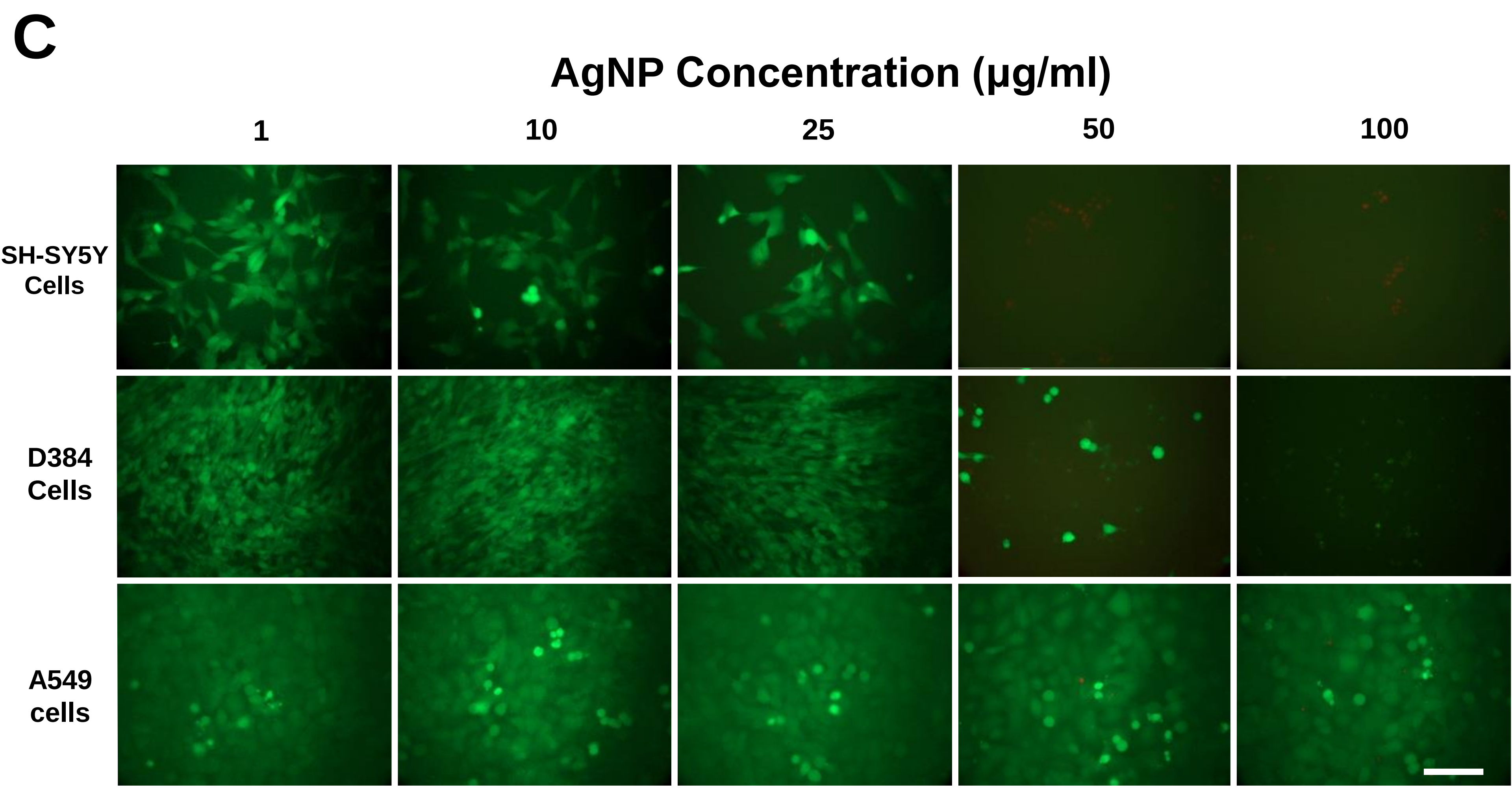
C
AgNP Concentration (µg/ml)
1
10
25
50
100
SH-SY5Y Cells
D384 Cells
A549 cells
